# Exploring the impact of number and type of comorbidities on the risk of severe COPD exacerbations in Korean Population: a Nationwide Cohort Study

**DOI:** 10.1186/s12890-021-01497-4

**Published:** 2021-05-06

**Authors:** Youngmee Kim, Ye-Jee Kim, Yu Mi Kang, Won-Kyung Cho

**Affiliations:** 1 Red Cross College of Nursing, Chung-Ang University, Seoul, Korea; 2 Department of Clinical Epidemiology and Biostatistics, Asan Medical Center, Seoul, Korea; 3 Department of Internal Medicine, Yale New Haven Health Bridgeport Hospital, Bridgeport, CT USA; 4 International Healthcare Center, Department of Pulmonary and Critical Care Medicine, Asan Medical Center, University of Ulsan College of Medicine, 88, Olympic-ro 43-gil, Songpa-gu, Seoul, 05505 Korea

**Keywords:** COPD, Exacerbation, Comorbidity

## Abstract

**Background:**

It is difficult to assess the impact of multiple comorbidities on clinical outcomes in chronic obstructive pulmonary disease (COPD). In this study, we aimed to investigate exacerbation-associated comorbidities, determine whether the number of comorbidities is an independent risk factor for exacerbation, and identify other exacerbation-associated factors in a Korean COPD population using a nationwide population-based cohort. This study focused on severe exacerbations that required hospitalisation or emergency room visits.

**Methods:**

The National Health Insurance Service-National Sample Cohort, version 2.0, data sampled between 2002 and 2015 were analysed. Data from two years after the diagnosis of COPD were analysed for each participant (N = 12,554, entire cohort). Moreover, 42% of the participants underwent additional health examinations (N = 5306, health-screening cohort). Fifteen comorbidities that were previously reported as risk factors for exacerbations were examined. A logistic regression model was used to analyse association with exacerbations.

**Results:**

Asthma (1.57 [1.39–1.76] and 1.24 [1.06–1.44]), lung cancer (1.84 [1.30–2.59] and 2.28 [1.54–3.37]), and heart failure (1.39 [1.16–1.67] and 1.52 [1.18–1.97]) were associated with exacerbation in both cohorts (odds ratio [95% confidence interval] in the entire cohort and health-screening cohort, respectively). The number of comorbidities was an independent risk factor, and old age, male sex, low body mass index, and current smoking were also independent risk factors. High cholesterol levels and body mass index exerted protective effects against exacerbation.

**Conclusions:**

The number of comorbidities, certain comorbidities such as asthma, lung cancer and heart failure, and low BMI were associated with an increased risk of severe exacerbation in COPD patients.

## Background

Chronic obstructive pulmonary disease (COPD) is a major global health concern. Medical comorbidities are prevalent among COPD patients [1] and have a notable impact on the clinical outcomes of COPD [2–4]. Previous studies have investigated the individual impact of comorbidities on COPD outcomes [5]. However, most patients with COPD have multiple comorbidities, some of which share risk factors, such as aging, smoking, or systemic inflammation, with COPD, while others have no evident pathophysiological relationship with COPD [3, 6]. Therefore, it has been challenging for researchers to consider the impact of multiple comorbidities when assessing the possible outcomes of patients with COPD.

A few disease-specific instruments are available to assess the burden of multiple comorbidities on the clinical outcomes of COPD. For example, the COPD-specific comorbidity test (COTE) was recently developed and validated to predict mortality [4]. Another tool, the COMorbidities in COPD (COMCOLD) index, is available to assess the effect of multiple comorbidities on the quality of life in COPD patients [7]. Putcha et al. reported that a simple count of comorbidities could predict exacerbation risk [8]. In our recent study, we also observed that the number of comorbidities might be an independent risk factor for COPD mortality [9].

Using a nationwide population-based cohort, the current study aimed to investigate the comorbidities associated with exacerbation, to determine whether the number of comorbidities could be an independent risk factor for exacerbation, and to identify other predictors associated with exacerbation in a Korean COPD population. The study focused on severe COPD exacerbations, which were defined as hospitalisations or emergency room visits due to worsening respiratory symptoms [1].

## Methods

### Study design, data sources, and participants

The Korean National Health Insurance Service (KNHIS), which covers approximately 97% of the nation’s population, has two components: mandatory social health insurance and medical aid. The medical aid program is a form of public support that uses government subsidies to provide healthcare services to low-income people. The KNHIS established the National Health Insurance Service-National Sample Cohort (NHIS-NCS) for research purposes, and it includes all medical information related to insurance claims. Detailed information on the KNHIS has been described elsewhere [9, 10].

This study used the NHIS-NCS version 2.0 (NHIS-NCS v2.0) data sampled between 2002 and 2015. The NHIS data are de-identified by the Korean government [11]. This study was reviewed and approved by the Institutional Review Board of Asan Medical Center (approval number: 2018-0971). The NHIS database did not include spirometry data or information on participants’ physical symptoms that are required for the diagnosis of COPD. Thus, COPD patients were recruited based on their International Classification of Disease-Tenth Revision (ICD-10) codes and prescription details.

Specifically, among the 1,108,369 participants in this cohort, patients were required to meet the following inclusion criteria, which are similar to those reported in a previous study [12]: ≥ 40 years of age; ICD-10 codes for COPD (J43–J44, except J430), and COPD medication use confirmed at least twice per year, which was assessed using prescription refill patterns from insurance claims data. The COPD medications considered in this study included long-acting muscarinic antagonists (LAMA), long-acting beta-2 agonists (LABA), inhaled corticosteroids (ICS), methylxanthines, and systemic beta-agonists.

**Fig. 1:**
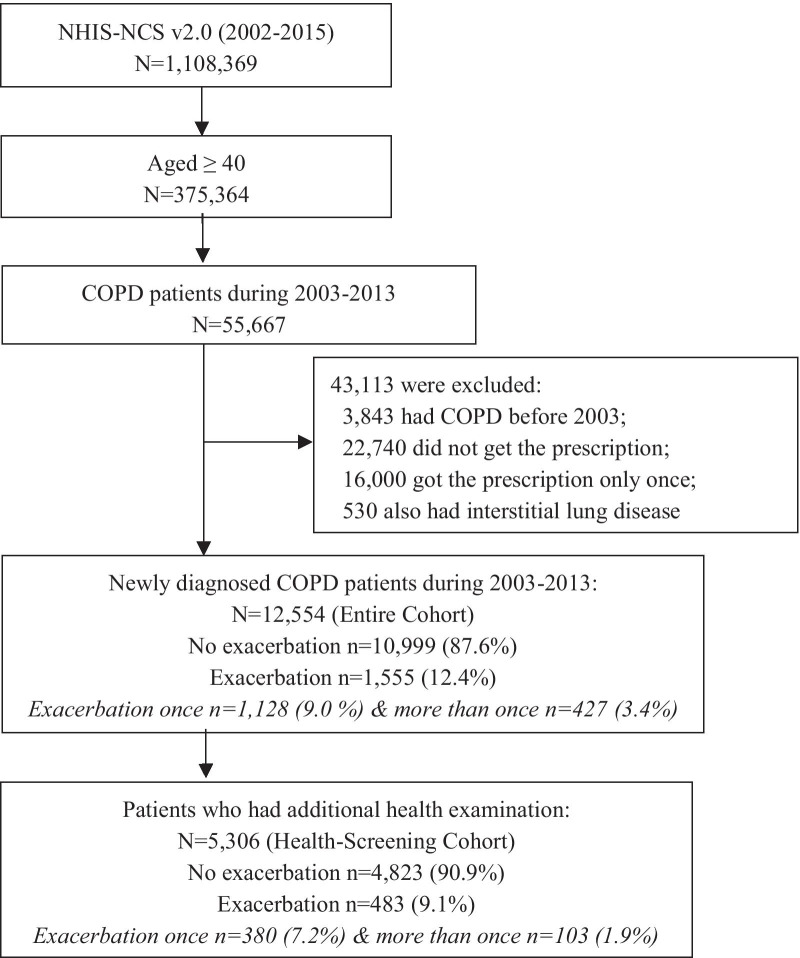
Flow diagram showing the study participants from the NHIS-NSC v2.0: the National Health Insurance Service-National Sample Cohort version 2.0

After allowing a 1-year pre-study period, newly diagnosed COPD patients were selected based on the abovementioned inclusion criteria. This was performed to avoid any potential diagnostic conflicts. Only the 2-year post-diagnosis data were analysed for each study participant to avoid differences in follow-up periods between patients (entire cohort). Some patients in the entire cohort underwent an additional national health examination if desired. Detailed information, including laboratory data, was available for these patients, and these data were analysed separately (health-screening cohort). Figure 1 represents the flow diagram of the selection process and number of study participants.

### Definitions

This study focused on severe exacerbations that required hospitalisation or emergency room visits. Episodes 7 days apart were considered as separate events [13]. Based on existing literature and data availability, data on 15 comorbidities previously reported as risk factors for exacerbations, clinical parameters, and sociodemographic variables were collected for the study [2, 14–17]. The 15 comorbidities were examined individually and grouped according to the affected organ system or disease mechanism [15] and each group was evaluated separately. Among the malignancy comorbidities, only malignancies that required hospitalisation for diagnosis or treatment were included to exclude the remote history of malignancies. The presence of target comorbidities was screened during the 1-year pre-study period using ICD-10 codes.

As for other variables, body mass index (BMI; kg/m^2^) was classified as follows: low (< 18.5), normal (18.5–22.9), overweight (23–24.9), and obese (≥ 25) [18]. The reference ranges of blood test findings, as recommended by the cohort user manual [11], were as follows: the normal haemoglobin level was 13.0–16.4 g/dL for males and 12.0–15.5 g/dL for females; the normal fasting blood glucose level was 100–125 mg/dL; the normal total cholesterol (TC) level was < 200 mg/dL; and the normal serum creatinine level was < 1.5 mg/dL.

### Statistical analysis

Baseline characteristics were compared between groups according to exacerbation history using a t-test and chi-squared test. All data are presented as mean ± standard deviation values for continuous variables or as frequencies and proportions for categorical variables. Multivariate logistic regression analyses were conducted to explore the associations between the variables and severe COPD exacerbations.

All variables for which the *p*-value was < 0.1, in the univariate analysis, were included in a multiple logistic regression analysis using the backward elimination method. The results were reported using adjusted odds ratios (ORs) and 95% confidence intervals (CIs). A multiple imputation procedure was applied using the Markov chain Monte Carlo method to impute missing values in the health-screening cohort. The multiple imputed datasets were analysed using the same analytical procedures, and the results from these analyses were combined to obtain an overall estimate. Data were analysed using SAS Enterprise Guide software version 7.1 (SAS Institute, Inc., Cary, NC, USA). Statistical significance was set at p < 0.05.

## Results

### Baseline characteristics of the entire and health-screening cohorts

Table 1 shows the baseline characteristics of the 12,554 eligible patients in the entire cohort according to the presence or absence of severe exacerbations. Overall, 54.8% of the patients were male, and the mean age was 66.74 years; 12.4% of patients experienced one or more severe exacerbations over the 2-year study period, and the mean number of severe COPD exacerbations was 1.49. The severe exacerbations group had a higher proportion of male patients, the mean age was significantly higher, and more patients received medical aid and had lower incomes compared to no exacerbation group.

**Table 1:** Baseline characteristics of the study participants: entire cohort

	Severe COPD exacerbation			*p*
	All N = 12,554 (100.0)	No N = 10,999 (87.6)	Yes N = 1555 (12.4)	
Severe COPD exacerbations, N (range)			1.49 ± 1.09 (1–11)	
General characteristics				
Male	6880 (54.8)	5867 (53.3)	1013 (65.1)	< 0.001
Age at the beginning of the study, yrs	66.74 ± 11.26	66.13 ± 11.20	71.05 ± 10.76	< 0.001
Health insurance type				
Medical aids	1681 (13.4)	1279 (11.6)	402 (25.9)	< 0.001
Health insurance	10,873 (86.6)	9720 (88.4)	1153 (74.2)	
Household income				
1st quintile	1723 (13.7)	1547 (14.1)	176 (11.3)	< 0.001
2nd quintile	1483 (11.8)	1318 (12.0)	165 (10.6)	
3rd quintile	1816 (14.5)	1633 (14.9)	183 (11.8)	
4th quintile	2471 (19.7)	2215 (20.1)	256 (16.)	
5th quintile	3265 (26.0)	2910 (26.5)	355 (22.8)	
Missing	115 (0.9)	97 (0.9)	18 (1.2)	
Comorbidities				
Comorbid diseases, N (range)	2.56 ± 1.88 (0–12)	2.52 ± 1.86 (0–12)	2.88 ± 2.01 (0–10)	< 0.001
0	1435 (11.4)	1307 (11.9)	128 (8.2)	< 0.001
1 or 2	5510 (43.9)	4866 (44.2)	644 (41.4)	
3 or 4	3569 (28.4)	3101 (28.2)	468 (30.1)	
≥ 5	2040 (16.3)	1725 (15.7)	315 (20.3)	
Cardiovascular comorbidity	6927 (55.2)	6028 (54.8)	899 (57.8)	0.026
Hypertension	5835 (46.5)	5098 (46.4)	737 (47.4)	0.439
Ischemic heart disease	1978 (15.8)	1695 (15.4)	283 (18.2)	0.005
Cardiac arrhythmia	888 (7.1)	749 (6.8)	139 (8.9)	0.002
Heart failure	943 (7.5)	763 (6.9)	180 (11.6)	< 0.001
Cerebrovascular disease	1273 (10.1)	1057 (9.6)	216 (13.9)	< 0.001
Peripheral vascular disease	1006 (8.0)	889 (8.1)	117 (7.5)	0.448
Respiratory comorbidity other than COPD	6917 (55.1)	5905 (53.7)	1012 (65.1)	< 0.001
Asthma	6667 (53.1)	5686 (51.7)	981 (63.1)	< 0.001
Bronchiectasis	738 (5.9)	611 (5.6)	127 (8.2)	< 0.001
Metabolic comorbidity	5840 (46.5)	5071 (46.1)	769 (49.5)	0.013
Dyslipidaemia	3352 (26.7)	2950 (26.8)	402 (25.9)	0.419
Diabetes mellitus	3078 (24.5)	2643 (24.0)	435 (28.0)	0.001
Osteoporosis	1914 (15.3)	1638 (14.9)	276 (17.8)	0.003
Chronic kidney disease	147 (1.2)	124 (1.1)	23 (1.5)	0.228
GI comorbidity	3625 (28.9)	3196 (29.1)	429 (27.6)	0.232
Gastroesophageal reflux disease	3213 (25.6)	2827 (25.7)	386 (24.8)	0.457
Chronic liver diseases	657 (5.2)	578 (5.3)	79 (5.1)	0.772
Malignancy comorbidity	478 (3.8)	381 (3.5)	97 (6.2)	< 0.001
Lung cancer	204 (1.6)	155 (1.4)	49 (3.2)	< 0.001
Stomach cancer	51 (0.4)	39 (0.4)	12 (0.8)	0.016
Colorectal cancer	47 (0.4)	44 (0.4)	3 (0.2)	0.211
Liver cancer	45 (0.4)	37 (0.3)	8 (0.5)	0.271
Thyroid cancer	41 (0.3)	37 (0.3)	4 (0.3)	0.609

Data are presented as N (%) or mean ± standard deviation, unless otherwise stated. *P*-values were obtained by the *t*-test or chi-squared test as appropriate; chronic liver diseases are liver cirrhosis or fatty liver disease. GI, gastrointestinal; COPD, chronic obstructive pulmonary disease

Regarding comorbidities, the mean number of comorbidities was 2.88 in the exacerbation group and 2.52 in the group without exacerbations. Only 11.9% of the patients without exacerbations and 8.2% of those with exacerbations showed no comorbidities. Hypertension, asthma, dyslipidaemia, diabetes mellitus (DM), and gastrointestinal reflux disease (GERD) were common comorbidities in this cohort. Comorbidities were more common in those with severe exacerbations than in those without.

Table 2 summarises the baseline characteristics of the 5306 patients in the health-screening cohort. In this cohort, 58.3% of the patients were male, and the mean age of the patients was 65.24 years. Moreover, 9.1% of the patients had severe exacerbation more than once during the study period; the mean number of severe exacerbation episodes over the study period in this group was 1.31. The severe exacerbation group comprised a higher proportion of males and older patients compared to no exacerbation group. The mean number of comorbidities was 2.89 in the severe exacerbation group and 2.51 in the group without exacerbations. Only 11.6% and 7.1% of those without and with exacerbations showed no comorbidities, respectively. Hypertension, asthma, dyslipidaemia, DM, and GERD were also common in this cohort. The presence of comorbidities was higher in those with severe exacerbations than in those without. TC level and BMI were lower in patients with severe exacerbations, and more current and former smokers were present in this group than in the other group.

**Table 2:** Baseline characteristics of the participants: health-screening cohort

	Severe COPD exacerbation			*p*
	All N = 5306 (100.0)	No N = 4823 (90.9)	Yes N = 483 (9.1)	
Severe COPD exacerbations, N (range)			1.31 ± 0.73 (1–6)	
General characteristics				
Male	3094 (58.3)	2725 (56.5)	369 (76.4)	< 0.001
Age at the beginning of the study, yrs	65.24 ± 10.46	64.82 ± 10.40	69.44 ± 10.13	< 0.001
Health insurance type				
Medical aids	72 (1.4)	62 (1.3)	10 (2.1)	0.155
Health insurance	5234 (98.6)	4761 (98.7)	473 (97.9)	
Household income				
1st quintile	838 (15.8)	759 (15.7)	79 (16.4)	0.650
2nd quintile	713 (13.4)	654 (13.6)	59 (12.2)	
3rd quintile	866 (16.3)	792 (16.4)	74 (15.3)	
4th quintile	1201 (22.6)	1095 (22.7)	106 (22.0)	
5th quintile	1551 (29.2)	1404 (29.1)	147 (30.4)	
Missing	65 (1.2)	57 (1.2)	8 (1.7)	
Comorbidities				
Comorbid diseases, N (range)	2.55 ± 1.82 (0–11)	2.51 ± 1.80 (0–11)	2.89 ± 1.99 (0–9)	< 0.001
0	596 (11.2)	562 (11.6)	34 (7.1)	0.004
1 or 2	2337 (44.0)	2132 (44.2)	205 (42.4)	
3 or 4	1535 (28.9)	1383 (28.7)	152 (31.5)	
≥ 5	838 (15.8)	746 (15.5)	92 (19.1)	
Cardiovascular comorbidity	2873 (54.2)	2603 (54.)	270 (55.9)	0.417
Hypertension	2405 (45.3)	2190 (45.4)	215 (44.5)	0.707
Ischemic heart disease	790 (14.9)	701 (14.5)	89 (18.4)	0.022
Cardiac arrhythmia	374 (7.1)	326 (6.7)	48 (9.9)	0.009
Heart failure	310 (5.8)	259 (5.4)	51 (10.6)	< 0.001
Cerebrovascular disease	432 (8.1)	380 (7.9)	52 (10.8)	0.027
Peripheral vascular disease	467 (8.8)	426 (8.8)	41 (8.5)	0.799
Respiratory comorbidity other than COPD	2892 (54.5)	2580 (53.5)	312 (64.6)	< 0.001
Asthma	2788 (52.5)	2486 (51.5)	302 (62.5)	< 0.001
Bronchiectasis	307 (5.8)	269 (5.6)	38 (7.8)	0.040
Metabolic comorbidity	2502 (47.2)	2258 (46.8)	244 (50.5)	0.120
Dyslipidaemia	1530 (28.8)	1390 (28.8)	140 (29.0)	0.939
Diabetes mellitus	1181 (22.3)	1053 (21.8)	128 (26.5)	0.019
Osteoporosis	800 (15.1)	728 (15.1)	72 (14.9)	0.913
Chronic kidney disease	39 (0.7)	34 (0.7)	5 (1.0)	0.397
GI comorbidity	1804 (34.0)	1645 (34.1)	159 (32.9)	0.599
Gastroesophageal reflux disease	1641 (30.9)	1499 (31.1)	142 (29.4)	0.446
Chronic liver diseases	285 (5.4)	253 (5.3)	32 (6.6)	0.200
Malignancy comorbidity	168 (3.2)	129 (2.7)	39 (8.1)	< 0.001
Lung cancer	76 (1.4)	51 (1.1)	25 (5.2)	< 0.001
Stomach cancer	14 (0.3)	9 (0.2)	5 (1.0)	0.006
Colorectal cancer	15 (0.3)	14 (0.3)	1 (0.2)	> 0.999
Liver cancer	12 (0.2)	8 (0.2)	4 (0.8)	0.019
Thyroid cancer	24 (0.5)	22 (0.5)	2 (0.4)	> 0.999
Health examination				
BMI, kg/m^2^	23.75 ± 3.38	23.88 ± 3.35	22.42 ± 3.44	< 0.001
Haemoglobin, g/dL	13.65 ± 1.57	13.65 ± 1.56	13.67 ± 1.65	0.741
Fasting blood glucose, mg/dL	102.24 ± 31.01	102.06 ± 30.04	104.08 ± 39.33	0.270
Total cholesterol, mg/dL	197.56 ± 49.95	198.4 ± 50.7	188.6 ± 40.9	< 0.001
Serum creatinine, mg/dL	1.01 ± 0.91	1.00 ± 0.88	1.13 ± 1.17	0.117

Data are presented as n (%) or mean ± standard deviation (SD), unless stated otherwise. *P*-values were obtained by the *t*-test or chi-squared test as appropriate; chronic liver diseases are liver cirrhosis or fatty liver disease. GI, gastrointestinal; COPD, chronic obstructive pulmonary disease

### Comorbidities and factors associated with severe exacerbations

**Fig. 2:**
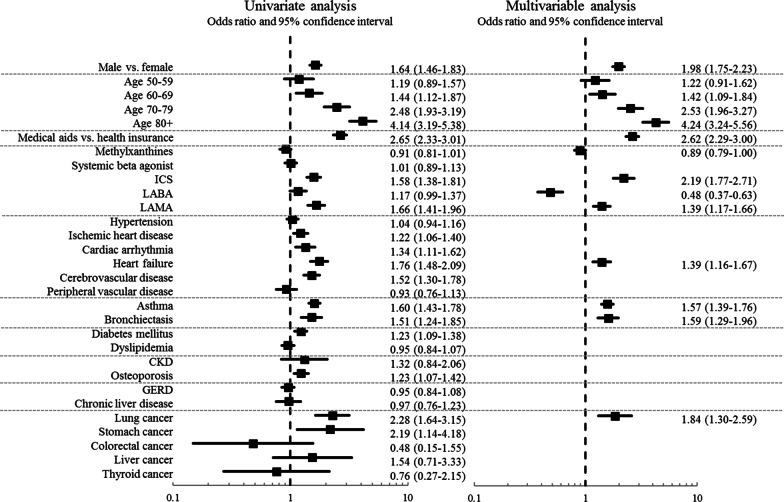
Factors associated with severe chronic obstructive pulmonary disease exacerbations in the entire cohort

Figure 2 presents various factors associated with severe COPD exacerbation in the entire cohort. While most of the variables were associated with severe exacerbations in the univariate analysis, the multivariate logistic analysis showed that only male sex, older age, receiving medical aid, and use of ICS and LAMA were independent risk factors for exacerbation. Among the comorbidities, heart failure, asthma, bronchiectasis, and lung cancer were the only factors significantly associated with severe exacerbations. In addition, the use of LABA was associated with reducing severe exacerbation.

**Fig. 3:**
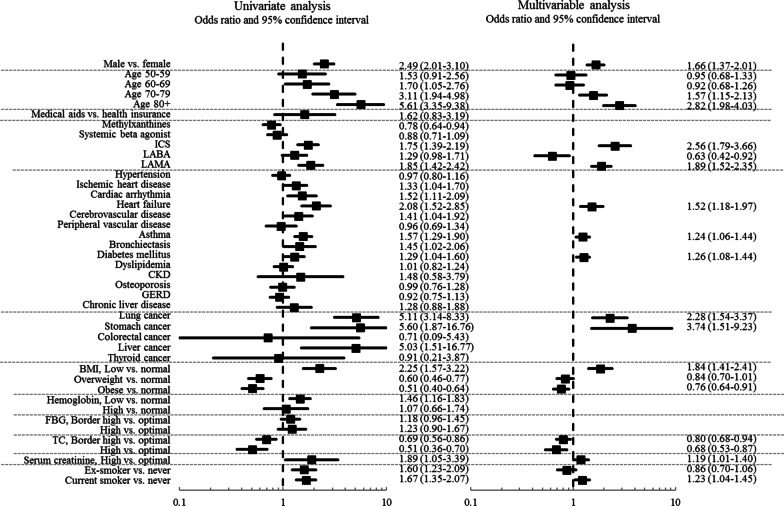
Factors associated with severe chronic obstructive pulmonary disease exacerbations in the health-screening cohort. The final multivariable model was adjusted for sex, age, health insurance type, COPD medication use (Methylxanthines, ICS, LABA & LAMA), heart failure, asthma, bronchiectasis, and lung cancer. CKD, chronic kidney disease; ICS, inhaled corticosteroid; LABA, long-acting beta-2 agonist; LAMA, long-acting muscarinic antagonist; SABA, systemic beta-agonist

Figure 3 shows the factors associated with severe exacerbations in the health-screening cohort. In the multivariable logistic analysis, male sex, older age, use of LAMA and ICS, low BMI, high serum creatinine level, and current smoking status were independent predictors of severe exacerbation of COPD. The use of LABA, high BMI, and high cholesterol levels showed a protective effect against exacerbation in the multivariable analysis. Among comorbidities, heart failure, asthma, DM, and lung and stomach cancer were significantly associated with severe COPD exacerbations.

### Impact of multimorbidity on severe exacerbations

Table 3 shows the significant associations between the number of comorbidities or comorbid groups and severe exacerbations in the entire cohort and the health-screening cohort, respectively. After adjusting for sex, age, health insurance type, and COPD medication use, when compared with patients with no comorbidities in the entire cohort, patients with ≥ 5 comorbidities and patients with five comorbid groups had a 1.40-fold and 2.20-fold higher risk of developing a severe exacerbation in the entire cohort, respectively (Table 3). In the health-screening cohort, when compared with patients with no comorbidity, patients with ≥ 5 comorbidities and patients with five comorbid groups had a 1.78-fold and 2.23-fold higher risk of developing a severe exacerbation, respectively, after adjusting for sex, age, COPD medication use, BMI, TC level, and smoking status (Table 3).

**Table 3:** Adjusted odds ratios for severe exacerbations of COPD relative to the number of comorbid diseases or comorbid groups in both cohorts

	Univariate analysis			Multivariable analysis for no. of comorbid diseases			Multivariable analysis for no. of comorbidity groups		
	OR	95% CI	*p*	OR	95% CI	*p*	OR	95% CI	*p*
Entire cohort*									
No. of comorbid diseases (range, 0–12)									
0	Ref		< 0.001	Ref		0.019			
1 or 2	1.35	(1.11 to − 1.65)		1.24	(1.01 to − 1.53)				
3 or 4	1.54	(1.25 to − 1.89)		1.35	(1.09 to − 1.68)				
≥ 5	1.87	(1.50 to − 2.32)		1.40	(1.12 to − 1.77)				
No. of comorbidity groups (range, 0–5)***									
0	Ref		< 0.001				Ref		0.001
1 or 2	1.34	(1.10 to − 1.62)					1.22	(1.00 to − 1.49)	
3 or 4	1.70	(1.39 to − 2.08)					1.44	(1.16 to − 1.77)	
5	3.26	(1.77 to − 5.99)					2.20	(1.16 to − 4.18)	
Health-screening cohort**									
No. of comorbid diseases (range, 0–12)									
0	Ref		0.004	Ref		0.002			
1 or 2	1.59	(1.09 to − 2.31)		1.63	(1.22 to − 2.17)				
3 or 4	1.82	(1.24 to − 2.67)		1.77	(1.31 to − 2.38)				
≥ 5	2.04	(1.36 to − 3.07)		1.78	(1.28 to − 2.46)				
No. of comorbidity groups (range, 0–5)***									
0	Ref		< 0.001				Ref		0.001
1 or 2	1.63	(1.13 to − 2.36)					1.68	(1.26 to − 2.23)	
3 or 4	1.96	(1.34 to − 2.87)					1.81	(1.35 to − 2.44)	
5	5.59	(2.08 to − 14.98)					2.23	(1.01 to − 4.93)	

CI, confidence interval; ICS, inhaled corticosteroid; LABA, long-acting beta-2 agonist; LAMA, long-acting muscarinic antagonist; Ref., reference

*The final multivariate models were adjusted for sex, age, health insurance type, and COPD medication use (methylxanthines, ICS, LABA, and LAMA)

**The final multivariable models were adjusted for sex, age, COPD medication use (ICS, LABA, LAMA), body mass index, total cholesterol, and smoking status

***Each comorbid disease was grouped according to the affected organ system or disease mechanism

## Discussion

We aimed to examine the comorbidities and other risk factors associated with severe exacerbation of COPD. We also checked the effect of the comorbidity number on severe COPD exacerbation among physician-diagnosed COPD patients. We observed that comorbidities such as asthma, lung cancer, and heart failure or low BMI and old age were associated with an increased risk of severe exacerbation in COPD patients. Our study also implicated that the number of comorbidities could be an independent risk factor of severe exacerbation of COPD.

Most of the study participants had comorbidities, and patients with a history of severe exacerbations had more comorbidities than patients without exacerbations (Tables 1 and 2). Though the difference was small, these findings are in agreement with previous studies [4, 14–16]. Further segmentation of the comorbidity numbers made it clear that the differences between the two groups (exacerbation and no exacerbation) occur owing to participants without any comorbidity and those with more than five comorbid diseases in both cohorts.

The multivariable logistic analysis revealed that only asthma, lung cancer, and heart failure were common comorbidities significantly associated with severe exacerbations of COPD in both cohorts (Figs. 2 and 3). Asthma has been reported to be a comorbidity that adversely affects the clinical outcome of COPD, including exacerbations [16, 17]. There are several similarities between asthma and COPD. Both are chronic airway inflammatory diseases even though inflammatory phenotypes are different, and both are characterized by mucous production and bronchoconstriction, though the reversibility of bronchoconstriction is different. Additionally, both can cause intermittent exacerbations of airway inflammation. Therefore, when combined, airway inflammation may perpetuate leading to a more severe airflow limitation [19]. It is not surprising that patients with both asthma and COPD may frequently experience severe exacerbations.

Among cardiovascular comorbidities, only heart failure was significantly associated with severe exacerbations in both cohorts. Heart failure has previously been shown to be an independent risk factor for adverse outcomes in patients with COPD [16, 20]. Heart failure often imposes diagnostic challenges since it can mimic an acute exacerbation of COPD. Since COPD and heart failure often precipitate each other’s exacerbations, it is not surprising that heart failure was found to be associated with severe COPD exacerbation in this study [20]. However, other cardiovascular comorbidities showed no significant association with exacerbation, which was unexpected given that cardiovascular comorbidity is a well-established factor associated with adverse clinical outcomes in patients with COPD [14, 21, 22]. Lung cancer was also an independent predictor of exacerbation, which is consistent with the results of previous studies [16, 23, 24]. Of note, it has been reported that COPD exacerbation tends to be more severe in the setting of lung cancer, and the presence of COPD is associated with worse outcomes in patients with lung cancer [25, 26].

Overall, the findings of our study support those of previous studies that have shown an association between key comorbidities and severe exacerbation of COPD. However, given that this study only examined comorbidities previously reported to be risk factors for poor clinical outcomes in COPD, the effect of comorbidities on severe exacerbation of COPD appears to be less evident in the current study. Notably, one previous study on the effect of chronic comorbidities on COPD exacerbations in the Korean population also found that of the 13 common chronic comorbidities evaluated in that study, only asthma was an independent risk factor for COPD exacerbation [17]. Taken together, these findings indicate that severe exacerbations of COPD might be less or differently affected by the presence of chronic comorbidities in the Korean population. However, a comparative study is required to confirm the ethnic differences in this area.

The impact of factors other than comorbidities was also assessed (Figs. 2 and 3). In this study, both cohorts showed that male sex was significantly associated with severe COPD exacerbation, unlike some previous studies [27]. Older age is also a widely accepted risk factor for COPD exacerbations [28], and this was confirmed in this study. In addition, low BMI contributed to exacerbations, and a high TC level protected against exacerbations in our study participants, suggesting a relationship with nutritional deficits. Indeed, previous studies have shown that low BMI is a risk factor for all-cause mortality in patients with COPD, and it is a well-established predictor of COPD exacerbation [28, 29]. As seen in previous study [28], the current study provided evidence that smoking cessation reduces exacerbations, emphasizing the importance of smoking cessation in the COPD population. Notably, the use of ICS and LAMA was associated with exacerbations, and the use of LABA was associated with fewer exacerbations in both cohorts. However, given the lack of a detailed history of the patients’ condition and other critical information, including pulmonary function data, it is difficult to interpret this finding.

Assessment of the impact of multimorbidity showed a significant association between the number of comorbidities or comorbid groups and severe exacerbations in both cohorts (Table 3). This indicates that the number of comorbidities or comorbid groups could be another independent contributor to severe exacerbations of COPD. Given that the majority of individual comorbidities were not seen to be independent predictors of exacerbation in this study, this finding is quite intriguing. These observations suggest that, first, some, but not all, comorbidities can have a direct impact on the development of severe exacerbations; second, comorbidities that were not independent risk factors for exacerbation may still contribute to the development of severe COPD exacerbations, possibly by increasing the total burden of comorbidities.

As discussed above, it is quite challenging to identify ways in which physicians can consider the impact of multiple comorbidities when assessing exacerbation risk. Recently, Putcha et al. reported a simple score using 14 comorbidities, in which a one-point increase in the comorbidity count was associated with a 21% higher exacerbation risk [8]. Although this comorbidity score was calculated without including some of the common comorbidities of COPD, such as lung cancer or asthma, this study suggested that a simple count of comorbidities could assess exacerbation risk. Our recently published study using the same NHIS-NCS v2.0 cohort also suggested that the number of comorbidities might be an independent risk factor for COPD mortality, especially all-cause mortality [9]. We believe that our study, as well as the study by Putcha et al., provide evidence supporting the use of number of comorbidities as a reasonable tool to assess the burden of multiple comorbidities given the lack of a proven evaluation tool.

Overall, our study suggested that some comorbidities associated with COPD could directly affect severe exacerbation of the disease. In contrast, though some comorbidities did not have any direct effect, they might indirectly influence COPD exacerbation, probably by increasing the total comorbidity burden, as the number of comorbidities was an independent risk factor for severe exacerbation. Therefore, the findings of our study are in line with those of previous studies that reported that comorbidities are important risk factors for COPD exacerbation.

The present study had some limitations. To diagnose COPD, pre- and post-bronchodilator spirometry data are needed. This is important to differentiate between asthma and COPD, and to assess the severity of COPD. Unfortunately, these data were not available for further analyses. It has been shown that one of the important predictors of frequent exacerbations is an exacerbation history in COPD [30], but we did not analyse the impact of the number of exacerbations in each patient separately as the aim of our study was to compare the effects of comorbidities between groups with and without exacerbations. We followed only newly diagnosed patients by physicians and addressed drug compliance by checking medication use. We believe that these measures minimised any potential bias. Furthermore, the current study examined only physician-diagnosed comorbidities, whereas most previous comorbidity studies used self-reported data, which may limit their internal validity [4, 31, 32]. This is a population-based study, and our study findings only apply to this set of population. Results should be further evaluated by performing external validation on diverse COPD populations.

## Conclusions

Of the 15 comorbidities reported to be associated with adverse outcomes of COPD, only 3, i.e., asthma, lung cancer, and heart failure, were found to be associated with an increased risk of severe exacerbation in this population of Korean patients with COPD. In addition, low BMI and old age were related to severe exacerbation in COPD patients. This study also observed that the number of comorbidities could be an independent risk factor for severe exacerbations. Further research is required to translate the current knowledge into standard patient-centred COPD care.

## Data Availability

All data generated or analysed during this study are included in this published article.

## Abbreviations

COPD: Chronic obstructive pulmonary disease
KNHIS: Korean National Health Insurance Service
NHIS-NCS: National Health Insurance Service-National Sample Cohort
ICD-10: International Classification of Disease-Tenth Revision
LAMA: Long-acting muscarinic antagonist
LABA: Long-acting beta-2 agonist
ICS: Inhaled corticosteroid
BMI: Body mass index
TC: Total cholesterol
CI: Confidence intervals
OR: Odds ratio
GERD: Gastrointestinal reflux disease (GERD)
DM: Diabetes mellitus
